# Supplementary material for: Unveiling phonons in a molecular qubit with four-dimensional inelastic neutron scattering and density functional theory
Source: Nat Commun. 2020 Apr 9;11:1751. doi: 10.1038/s41467-020-15475-7 (PMC7145838; doi:10.1038/s41467-020-15475-7)
Supplement: Supplementary file 1 — Supplementary Information [file 41467_2020_15475_MOESM1_ESM.pdf]

Supplementary Information

Unveiling phonons in a molecular qubit with four-dimensional inelastic neutron scattering and density functional theory

Garlatti et al.

Supplementary Figure 1 – Bragg Peaks

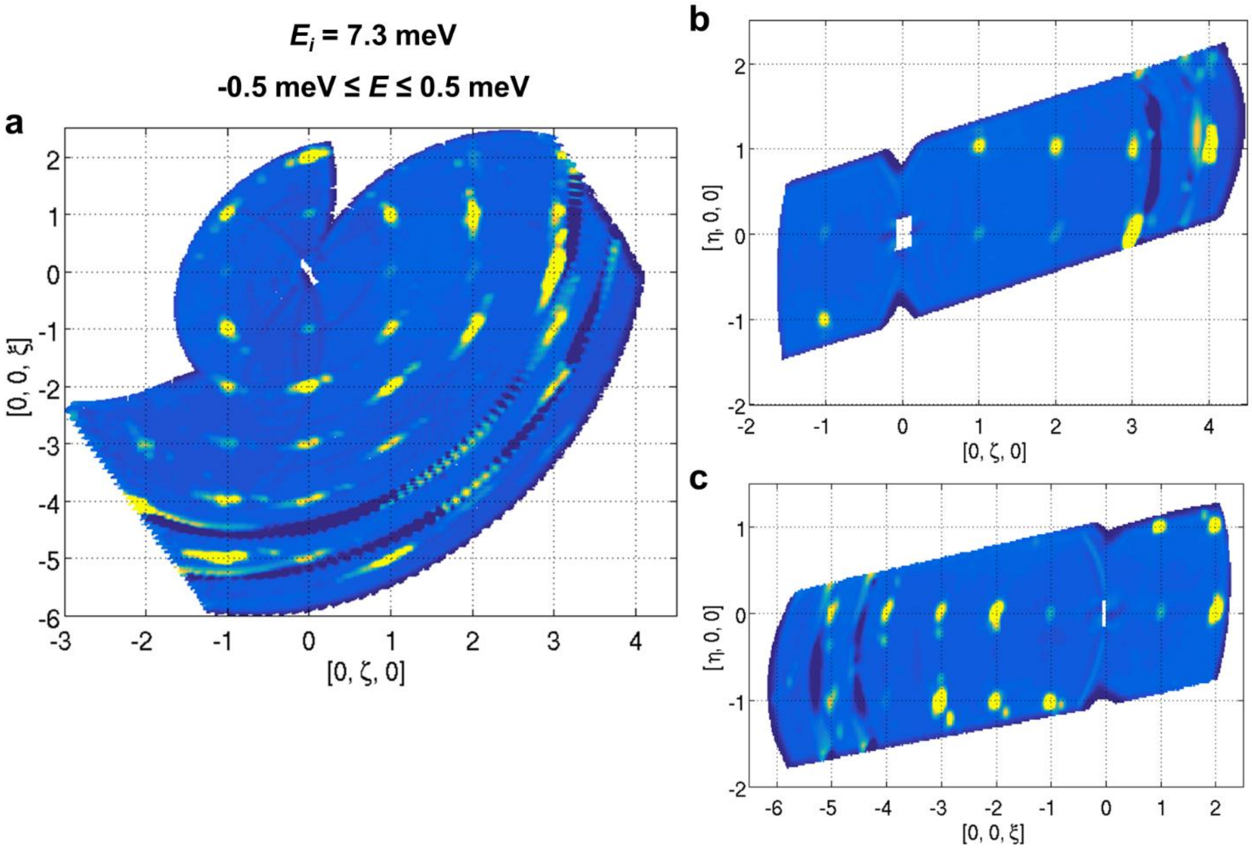

Supplementary Figure 1. Bragg peaks in the E = 0 plane. Explored portions of the reciprocal space allowed by LET dynamical range when E_i =7.3 meV, obtained integrating LET data in an energy range of ±0.5 meV around E = 0. The largest explored area is in the [0, ζ, 0] – [0, 0, ξ] plane (**a**), reflecting the alignment of the [VO(acac)$_2$] crystals with the vector **a***perpendicular to the scattering plane. Smaller portions are allowed by the experimental setup in the [η, 0, 0] direction, corresponding to the out-of-plane detector coverage (**b**, **c**). Bragg peaks are evident in all the panels, in correspondence of integer values for Miller indexes. This demonstrate the correct alignment of all the [VO(acac)$_2$] crystals. All directions are expressed in terms of reciprocal lattice vector units.

Supplementary Figure 2 – Spin-phonon couplings along Γ-Z

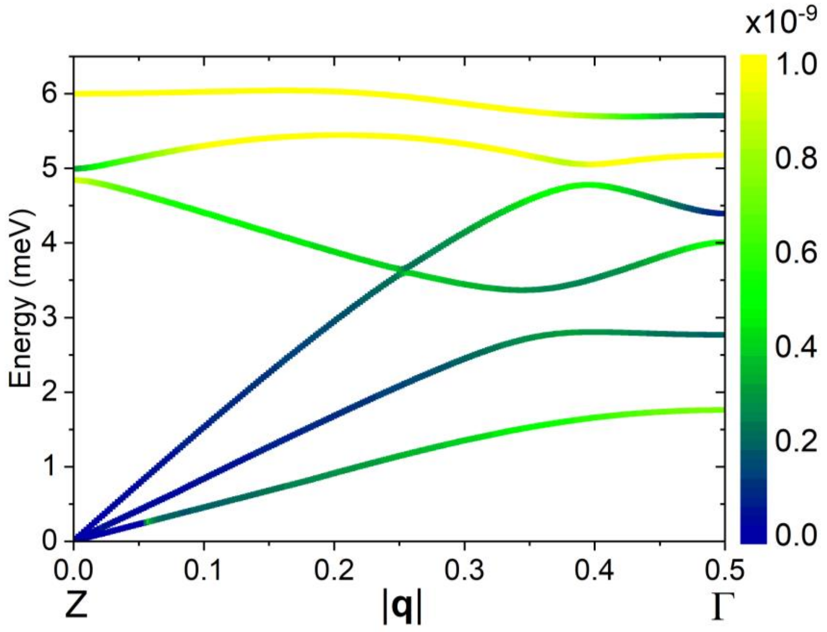

Supplementary Figure 2. DFT spin-phonon couplings. Colours map spin-phonon couplings squared norm $V^2_{\mathrm{SPh}}(\omega_j,\mathbf{q})$ calculated as in equation (1) of the main text for the low-energy phonon modes along Γ-Z, as a function of $|\mathbf{q}|$. At the ACs the strong coupling of the optical modes is "transferred" to the acoustic ones.

Supplementary Note 1 – Spin-phonon couplings: comparison with an isolated molecule

In order to compare the magneto-vibrational coupling coefficients of the isolated molecule and the spin-phonon ones of the molecular crystal some care must be used. According to their definition, the spin-phonon coupling coefficients present a dependence $1/\sqrt{N_q}$, where N_q is the number of q-points used [1]. This makes it possible for the spin relaxation time, which depends on the square of the spin-phonon coupling coefficients, to converge as an integral over the Brillouin zone. Therefore, when comparing the coupling coefficients of single modes obtained from calculations with a different total number of q-points, a rescaling must be applied. According to the same principle, an additional factor of 2, equal to the number of molecules in the unit cell, is present between the square of the coefficients relative to the crystal Γ point and the isolated molecule. Table S1 contains the isolated molecule magneto-vibrational coupling coefficients rescaled by a factor 500, in accordance with the fact we integrated the Γ-X direction of the Brillouin zone with 250 q-points.

Table S1 also compares the single-molecule energies and the magneto-vibrational couplings with the energies and the spin-phonon couplings obtained for the single crystal. Clearly, vibrational modes of an isolated molecule do not show dispersion and can only be compared with single crystal ones at the Γ point**.** The comparison shows a substantial shift to lower energies of the first molecular mode with respect to the first phonon frequency at Γ (from about 4.8 meV to 3 meV), corroborating the non-negligible effect of crystal packing already at the level of a Γ-point description of the phonons. There is a good correspondence between the molecular displacements associated with the low-energy isolated-molecule modes and those of optical

phonons at the Γ point, leading to similar values of the calculated magneto-vibrational coupling coefficients with respect to spin-phonon ones.

As far as relaxation is concerned, it is important to remark that while the crystal provides a continuous spectrum of phonons at low energies, the isolated molecule does not. This implies that, starting from isolated-molecule vibrational modes and considering direct processes, it is not possible to predict a finite spin coherence time, unless a strict resonance condition is reached between the molecule energy gaps and the accessible vibrational modes. Thus, this condition prevents direct processes to occur for reasonable values of the applied magnetic fields, suppressing the main relaxation mechanism and yielding longer coherence times for the isolated molecules than in single crystal.

**Phonon energies at the Γ point Crystal (meV)**	**$V^2_{SPh}(\omega_j)$ at the Γ point**	**Vibrational energies Single molecule (meV)**	**$V^2_{mv}(\omega_j)$**
4.83976	$9.02935 \cdot 10^{-10}$	3.0274	$1.14589 \cdot 10^{-9}$
4.99128	$3.48216 \cdot 10^{-10}$	3.77901	$2.41544 \cdot 10^{-10}$
5.99873	$1.61404 \cdot 10^{-9}$	6.61085	$2.66206 \cdot 10^{-9}$
7.32339	$3.53055 \cdot 10^{-9}$	6.95946	$1.58282 \cdot 10^{-10}$
7.63817	$2.0685 \cdot 10^{-9}$	7.00635	$3.59501 \cdot 10^{-9}$
7.68574	$5.52563 \cdot 10^{-10}$	7.44992	$1.60538 \cdot 10^{-10}$

Supplementary Table 1. Comparison with an isolated molecule. Calculated energies of the low-energy optical phonon modes of the crystal at the Γ point (calculated with the supercell method plus the 13% down-scaling, see Methods section of the main text) compared with the lowest vibrational energies for an isolated molecule (with the same 13% down-scaling). The table also reports the corresponding magneto-vibrational couplings squared norms $V^2_{mv}(\omega_j)$ compared to the spin-phonon coupling coefficients squared norm $V^2_{SPh}(\omega_j)$ calculated as in equation (1) of the main text (taking into account the normalization for the q-sampling).

References

[1] A. Lunghi and S. Sanvito, *Sci. Adv.* **5**, eaax7163 (2019).
